# The experiences of those affected by parental young onset dementia: A qualitative systematic literature review

**DOI:** 10.1177/1471301220988231

**Published:** 2021-01-30

**Authors:** Anna V Cartwright, Charlotte R Stoner, Richard D Pione, Aimee Spector

**Affiliations:** Department of Clinical, Educational and Health Psychology, 4919 University College London (UCL), UK

**Keywords:** young onset dementia, lived experience, qualitative, children, family carers

## Abstract

**Aim:**

To develop understanding of the lived experiences of children of people living with young onset dementia, defined as individuals both under and over the age of 18 years whose parent was diagnosed with dementia before the age of 65 years.

**Method:**

A critical appraisal and thematic synthesis of the available qualitative literature regarding the lived experience of individuals whose parent has a diagnosis of young onset dementia. A three-stage approach for conducing thematic synthesis was followed.

**Results:**

15 articles were included in the review. Four analytical themes and 11 subthemes were found. The analytical themes were ‘making sense of dementia’, ‘impact of dementia’, ‘coping’ and ‘support’.

**Conclusions:**

The experiences of those affected by parental young onset dementia vary widely. There is a lack of knowledge and understanding of young onset dementia by professionals and the public, and a scarcity of appropriate support. This has clinical implications for professionals working with families affected by young onset dementia, in particular with regards to service design and delivery.

## Introduction

### Dementia and young onset dementia

Young onset dementia is defined as the presentation and diagnosis of dementia before the age of 65 years. This is a widely used but arbitrary and socially determined cut-off point, possibly originating from retirement age (Rossor et al., 2010). There has been inconsistency in terminology used within the literature, with ‘young onset’, ‘early onset’ (Johannessen & Moller, 2013), ‘working age’ (Rudman et al., 2011) and ‘presenile’ used interchangeably. Recently, the term ‘young onset dementia’ has become the most frequently used (Koopmans & Rosness, 2014).

There are approximately 42,000 people in the United Kingdom with a diagnosis of young onset dementia, representing around 5% of the total number of people living with dementia (Dementia U.K., 2014). The most common causes of dementia in both older and younger people are Alzheimer’s disease (AD), vascular dementia, frontotemporal degeneration (FTD) and dementia with Lewy bodies (Rossor et al., 2010).

### Differences associated with young onset dementia

People living with young onset dementia are more likely to have dementias other than AD, such as FTD, characterised by changes in personality, behavioural disturbances and reduced empathy and motivation (Jefferies & Agrawal, 2018). The younger the onset, the more likely it is that dementia is caused by a genetic or metabolic disease (Sampson et al., 2004).

Differences in presentation can complicate the diagnostic process and lead to misdiagnosis. For example approximately one-third of people with young-onset sporadic AD have non-amnesic deficits, compared to only 5% of those with later-onset variants (Koedam et al., 2010). Neuropsychiatric symptoms, such as aggression, agitation, depression, anxiety, hallucinations, delusions, disinhibition and apathy are more common (Mendez, 2006). As a result, neurodegenerative disorders are often misdiagnosed as psychiatric disorders. For example one review reported that 28.2% of people with a neurodegenerative disorder had received a prior psychiatric diagnosis, and in people with behavioural variant frontotemporal dementia, younger age significantly increased the rate of prior psychiatric diagnosis (Woolley et al., 2011). There are also more varied differential diagnoses, many of which are infections, toxic-metabolic or inheritable conditions. Difficulties obtaining a timely and accurate diagnosis are therefore more common for people living with young onset dementia, as symptoms are often attributed to other causes.

### Impact of young onset dementia

Adults diagnosed with young onset dementia commonly have a range of important roles and responsibilities, including employment, parenting, caring for elderly family members and significant financial commitments. They tend to be more physically fit at the time of diagnosis, with fewer comorbid health problems than those diagnosed in later life. Dementia is commonly perceived as an illness of old age, and people living with young onset dementia often report the diagnosis as a shock, with many experiencing adjustment difficulties (Sansoni et al., 2016).

Common issues raised by people living with young onset dementia in the early stages include difficulties being taken seriously by doctors and obtaining a diagnosis (Johannessen & Moller, 2013). In the post-diagnostic period, there are often significant changes to the person’s lifestyle, including withdrawal from activities such as working, driving, hobbies and socialising (Spreadbury & Kipps, 2019). These changes can lead to strain on relationships, social isolation and feelings of marginalisation, increased financial pressure, poor self-esteem and reduced sense of purpose (Harris, 2004; Roach & Drummond, 2014). Dementia can have a significant impact on a person’s identity as a worker, partner and parent (Clemerson et al., 2014). For some, the experience of dementia can also have a positive impact, for example the relationship with their caregiver may be ‘closer’ or ‘strengthened’ (Harris, 2004; Johannessen & Moller, 2013).

### Caring for a person living with young onset dementia

Many people living with dementia are cared for at home by a relative or friend (Newbronner et al., 2013). Spouses and adult children are typically the main sources of informal care (Wawrziczny et al., 2016). Caring responsibilities may include emotional support, practical support with tasks such as cooking and cleaning, personal care, such as washing and toileting, and support with finances.

Spouses report difficulties managing the behavioural and psychological symptoms of dementia, experiencing grief associated with the ‘loss’ of their spouse and finding it hard to balance the caring role with other responsibilities (Sansoni et al., 2016).

The responses to caregiving are thought to differ for children and young people, suggesting that findings from studies with spouses and other family members may not apply to this population. Spreadbury and Kipps (2019) reported that adolescent and young adult caregivers may be more susceptible to mental health problems as a result of caregiving and were reported to use different coping strategies. Young people have also reported learning new skills, feeling useful and feeling a sense of closeness to the family as a result of caregiving (Joseph et al., 2012).

### Professional support

Typically, people presenting with young onset dementia are referred to dementia services set-up for older adults and receive their diagnosis and care from old age psychiatrists (The Royal College of Psychiatrists, 2018). However, staff may be less well equipped to provide specialist support and advice catering to the particular needs of people living with young onset dementia and their families (Sansoni et al., 2016). Richardson et al. (2016) emphasised the need for the development of interventions that benefit people living with young onset dementia and their carers. Reviews have highlighted the lack of clear diagnostic pathways, poor availability of relevant information, lack of appropriate referrals to support services and paucity of age-appropriate services (Sansoni et al., 2016; Spreadbury & Kipps, 2019).

### Rationale and aims

In this review, the term ‘children’ is used to refer to offspring, including those both under and over the age of 18 years, or stepchildren. There are a small number of reviews that have included studies reporting the experiences of children of people living with young onset dementia (Cabote et al., 2015; Millenaar et al., 2016; Sansoni et al., 2016; Spreadbury & Kipps, 2019; Svanberg et al., 2011; Van Vliet et al., 2010). However, none of these have focused exclusively on the children. This is likely due to the large gap in research into their needs and experiences, which has been previously highlighted in the literature (Richardson et al., 2016). Over the past few years, there has been an increase in the number of studies published in this field but there has not been a recent systematic review of the literature specifically exploring the experiences of individuals whose parent has a diagnosis of young onset dementia.

The aim was to answer the research question: What are the lived experiences of individuals whose parent has been diagnosed with young onset dementia? This question was kept broad to allow for the inclusion of studies focussing on different aspects of lived experience for both children under the age of 18 years and adult children. These findings will lead to a greater understanding of the children’s experiences, which can be used to inform service development, ensuring the needs of families affected by young onset dementia are better met.

## Methods

### Search strategy

Four electronic databases were searched on September 13, 2019: PsycINFO, Ovid (MEDLINE), Cumulative Index to Nursing and Allied Health Literature and Embase. An example search strategy is included (Table 1). Searches were rerun on March 23, 2020 and results screened for potentially eligible studies. For each database, equivalent database-controlled terms were entered, with the search terms categorised into 4 groups: (1) Time of onset, (2) Condition, (3) Population and (4) Experience/qualitative approach. Search terms were combined using AND/OR linking operations. Search strategies consisted of Medical Subject Heading terms and keywords. Due to the relative scarcity of research, no date or age restrictions were imposed to ensure inclusion of all relevant articles.

**Table 1.** Example electronic search strategy conducted in PsycInfo.

Search concept	Search terms
1. Time of onset	‘working age*' OR ‘young* people' OR ‘earl* onset' OR ‘under 65*' OR ‘ young* onset' OR presenile
2. Condition	dement* OR alzheimer* OR *exp Presenile Dementia/*OR *exp Dementia/*OR *exp Alzheimer's Disease/*
3. Population	relative* OR child* OR family OR families OR son* OR daughter* OR care* OR *caregivers/*
4. Experience	experience* OR perception* OR perspective* OR impact* OR interview* OR qualitative*

*Note.* Medical Subject Heading (MeSH) terms used are reported in italics.

### Study selection

Search results were imported into EndNote reference management software. After removing duplicates, titles and abstracts were screened against the following criteria:

### Inclusion criteria

1. Published in English in a peer-reviewed journal.
2. Study population consisted primarily of the children (including those both under and over the age of 18 years and stepchildren) of people living with young onset dementia (defined as dementia diagnosed before the age of 65 years), or the data from children could be separated from that of other participants.
3. Stated aim of the study concerned individual experiences, caring experiences or implications of caring on the children.
4. Qualitative or mixed methods, primary research.

### Exclusion criteria

1. Published in non–peer-reviewed journals, grey literature or were unpublished theses.
2. Not an empirical article (e.g. was a review, conference abstract or protocol).
3. Solely quantitative data collected.

Full texts were sought for all articles thought to potentially meet the above criteria and screened against these criteria. Those considered borderline were discussed with AS in order to reach agreement. Supplementary searches were conducted, including searching reference lists of included articles, reference searching relevant systematic reviews and searching Google Scholar. Although some qualitative reviews only include as many articles required for conceptual saturation, there is no established method for reaching this point of saturation. We, therefore, included all studies meeting criteria.

### Data extraction

A standardised data extraction form was developed, and data extracted from all studies under the following headings: study and country, research question/aim, sample, age of participants at time of interview, data collection and approach to analysis. When these data were not reported, authors were contacted for further information.

### Quality appraisal

Quality appraisal was conducted using the Critical Appraisal Skills Programme (CASP) qualitative checklist (Critical Appraisal Skills Programme (CASP) UK, 2018). This outlines 10 questions to help the reviewer appraise qualitative studies with regards to their validity, findings and value. Most questions require a ‘yes’, ‘no’ or ‘cannot tell’ response. CASP was chosen, as it is reported to be the most commonly used tool in the quality appraisal process of qualitative evidence syntheses (Majid & Vanstone, 2018). Only studies considered methodologically ‘flawed’ were excluded (Dixon-Woods et al., 2004).

### Data synthesis

A three-stage thematic synthesis approach was used, adopting methods described by Thomas and Harden (2008). This approach was chosen as it addresses review questions, with the aim of informing clinical practice. Other qualitative synthesis methods, such as meta-ethnography, are more suitable for developing new theories or models (Noblit & Hare, 1988).

The findings from each article were entered into NVivo software and text was coded ‘line-by-line’ according to its meaning and content, enabling the translation of concepts from one study to another in an iterative fashion. For example the line ‘When I got told he had dementia I was just so shocked and didn’t know what to do...’ (Hall & Sikes, 2017) was initially coded ‘shock of diagnosis’.

The second stage involved examining similarities and differences between initial codes, grouping them into a hierarchical structure and assigning descriptive codes to capture the meaning of these groups. This stage was conducted independently by RP and AC and discussed, to decide upon a final hierarchical structure. As an example, the code ‘shock of diagnosis’ was initially grouped under the descriptive code ‘diagnostic process’. Other initial codes grouped within ‘diagnostic process’ included ‘importance of getting a diagnosis’ and ‘seeking diagnosis’.

The final stage of synthesis involved using descriptive codes to develop analytical themes, going beyond the content of the original studies to answer the review questions and inform clinical practice. For example in this final stage of synthesis, the descriptive code ‘diagnostic process’ was included under the subtheme ‘change over time’, within the broader analytic theme, ‘making sense of dementia’.

## Results

**Figure 1.**
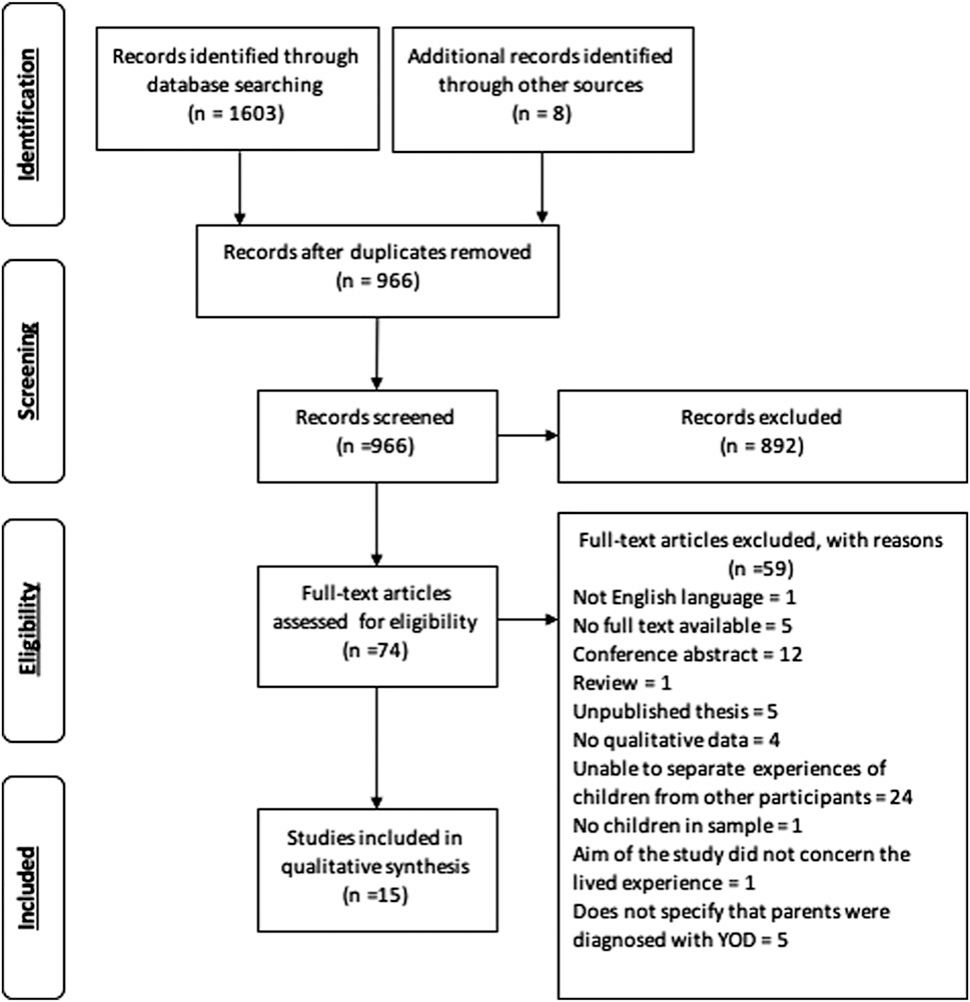
PRISMA (Moher et al., 2009) flow diagram showing the screening process. YOD: young onset dementia.

The initial search identified 1603 studies. 15 articles were included in the final review, after removing duplicates and those not meeting criteria. Figure 1 shows the number of articles excluded at each stage. If an article failed to meet multiple criteria, the primary reason for exclusion was noted. The 15 articles represented findings from 10 unique studies, as some articles reported findings from the same research projects.

### Quality appraisal

All studies were considered to be of satisfactory quality to be included. A summary of the information extracted using the CASP checklist is presented in Table 2.

**Table 2.** Quality appraisal using Critical Appraisal Skills Programme qualitative checklist.

Study	Aim	Method	Design	Recruitment	Data collection	Relationship	Ethical issue	Analysis	Finding	Value
Allen and Oyebode (2009)	✓	✓	✓	✓	✓	-	✓	✓	✓	✓
Svanberg et al. (2010)	✓	✓	✓	✓	✓	✗	✓	✓	✓	✓
Nichols et al. (2013)	✓	✓	✓	✓	-	-	-	✓	✓	✓
Barca et al. (2014)	✓	✓	✓	✓	✓	✗	✓	✓	✓	✓
Millenaar et al. (2014)	✓	✓	✓	✓	✓	✗	-	✓	✓	✓
Johannessen et al. (2015)	✓	✓	✓	✓	✓	✗	✓	✓	✓	✓
Hutchinson, Roberts, Daly et al. (2016)	✓	✓	✓	✓	✓	✗	✓	✓	✓	✓
Hutchinson, Roberts, Kurrle et al. (2016)	✓	✓	✓	✓	✓	✗	✓	✓	✓	✓
Johannessen et al. (2016)	✓	✓	✓	✓	✓	✗	✓	✓	✓	✓
Hall and Sikes (2017)	✓	✓	✓	✓	✓	✗	✓	✓	✓	✓
Sikes and Hall (2017)	✓	✓	✓	✓	✓	✓	✓	-	✓	✓
Gelman and Rhames (2018)	✓	✓	✓	✓	✓	✗	✓	✓	✓	✓
Hall and Sikes (2018)	✓	✓	✓	✓	✓	✗	✓	-	✓	✓
Sikes and Hall (2018)	✓	✓	✓	✓	✓	✓	✓	-	✓	-
Aslett et al. (2019)	✓	✓	✓	✓	✓	✗	✓	✓	✓	✓

*Note.* ✓ = criteria met, - = cannot tell or criteria partly met, ✗ = criteria not met.

All studies stated their aims and used appropriate methodology, research design and recruitment strategy. Most used semi-structured interviews, collecting data in a way that addressed the research question and included sufficient justification and detail as to how these were conducted. Nichols et al. (2013) did not provide enough information regarding methods of data collection to enable rating of this item.

No studies stated their epistemological position and very few considered the relationship between researcher and participants, apart from Sikes and Hall (2017, 2018), who discussed possible influences of personal experience. Most studies included sufficient detail regarding ethical considerations. Millenaar et al. (2014) and Nichols et al. (2013) provided limited information regarding this, stating that consent was sought, but did not discuss other issues, such as ethical approval or debrief.

All studies stated using a qualitative method of analysis, although some studies provided little detailed description regarding how exactly the analysis was conducted (Sikes & Hall, 2017, 2018). All studies used quotes to illustrate key findings and themes and discussed findings in relation to the research question. Due to overlap in findings and implications between the studies by Sikes and Hall (2017, 2018), the ‘value of research’ criterion of the later study was considered ‘partly met’.

### Selected studies

Table 3 provides a summary of included studies. All studies included by Sikes and Hall reported on findings from the same project, with three additional participants recruited between the start and end of the project. The two studies by Johannessen and colleagues and those by Hutchinson and colleagues also reported on findings from the same participants.

**Table 3.** Details of included studies.

Study and country	Research question/aim	Sample (M: F)	Age at interview in years (*M, SD*)	Data collection and approach to analysis
Allen and Oyebode (2009), UK	To explore the impact on young people’s well-being of having a parent with YOD.	12 children (5:7) of men with YOD, from 7 families	11–24 (19, 3.08)	Interviews, grounded theory
Svanberg et al. (2010), UK	To discover the experiences of the children of younger people with dementia and explore the impact of the diagnosis	12 children (6:6) of people living with YOD, from 9 families	11–18 (14.6, -)	Semi-structured interviews, grounded theory
Nichols et al. (2013), US and Canada	To learn about the experiences of children of people with FTD, what they had needed at various points in the patient's diagnostic process and course of illness	14 young people (4:10) caring for family member (8 fathers, 2 mothers, 2 stepfathers and 2 grandfathers) with FTD (defined as having onset before age 65 years)	8–18 (14.3, 3.00)	2 focus groups using semi-structured interview schedule, thematic analysis
Barca et al. (2014), Norway	To explore how adult children of a parent with YOD have experienced the development of their parents' dementia and what needs for assistance they have	14 children (2:12) of people living with YOD.	20–37 (-, -)	Semi-structured interviews, Corbin and Strauss’ (2008) reformulated grounded theory
Millenaar et al. (2014), Netherlands	To explore the experiences of children living with a young parent with dementia with a specific focus on the children’s needs	14 children (6:8) of people living with YOD, from 11 families	15–27 (21, -)	Semi-structured interviews, inductive content analysis
Johannessen et al. (2015), Norway	To explore how adult children of persons with YOD describe their experiences in everyday life with metaphors, and how these metaphors might be understood	14 children (5:9) of people living with YOD	18–30 (24, 4.00)	Semi-structured interviews, Steger’s (2007) three-step metaphor analysis
Hutchinson, Roberts, Daly et al. (2016), Australia	To explore the lived experiences of young people having a parent with YOD from the perspective of the social model of disability and to explore influencing factors that could enable these young people to be included and supported within their community	12 children (1:11) of people living with YOD	10–33 (24.2, 5.84)	Semi-structured interviews, thematic analysis using framework analysis
Hutchinson, Roberts, Kurrle et al. (2016), Australia	To explore the lived experience of young people living with a parent with YOD from the perspective of the social model of disability	12 children (1:11) of people living with YOD	10–33 (24.2, 5.84)	Semi-structured interviews, thematic analysis using framework analysis
Johannessen et al. (2016) Norway	To explore how adult children experienced the influence of their parents’ dementia on their own development during adolescence	14 children (5:9) of people living with YOD	18–30 (24, 4.00)	Semi-structured interviews, Corbin and Strauss’ (2008) reformulated grounded theory
Hall and Sikes (2017), UK	To give ‘voice to silenced lives’ and explore social and cultural experiences of having a parent with dementia	22 children of people with dementia (all but 1 diagnosed before age 65)	6–31 (20.9, 6.65)	Invited participants to ‘tell me your story’, auto/biographical approach, thematic analysis
Sikes and Hall (2017), UK	To address the gap in research and literature around living with dementia by focussing on the perceptions and experiences of children and young people who have a parent with YOD.	22 children of people with dementia (all but 1 diagnosed before age 65)	6–31 (20.9, 6.65)	Invited participants to ‘tell me your story’, auto/biographical approach, thematic analysis
Gelman and Rhames (2018), USA	To ask children and well-parents about the impact of living at home with a parent with YOD in order to better understand their experience and more effectively respond to their unique needs	8 children (3:5) of people living with YOD. Sample also included 4 of their mothers	15–20 (18, 1.85)	Semi-structured interview, thematic analysis
Hall and Sikes (2018), UK	To gain a sense of how individuals with different biographies go through similar social and cultural experiences: In this case, being a young person with a parent who has dementia	20 children (4:16) of people with dementia (all but 1 diagnosed before age 65)	7–31 (22.3, 5.14)	Interviews, thematic approach
Sikes and Hall (2018), UK	To collect in-depth, personal stories of children and young people who have or have had a parent with dementia	19 children (3:16) of people with dementia (all but 1 diagnosed before age 65)	8–31 (22.3, 5.28)	Invited participants to ‘tell me your story’, auto/biographical, life history approach
Aslett et al. (2019), UK	To explore the personal meaning attached to having a parent with YOD; to consider how this impacts on relationships with other family members; and to consider positive as well as negative impact of having a parent diagnosed with YOD.	5 children (2:3) of people living with YOD	23–36 (31.4, 6.02)	Semi-structured interviews, IPA

IPA: interpretative phenomenological analysis; FTD: frontotemporal degeneration; YOD: young onset dementia; ‘-‘: data not available.

**Figure 2.**
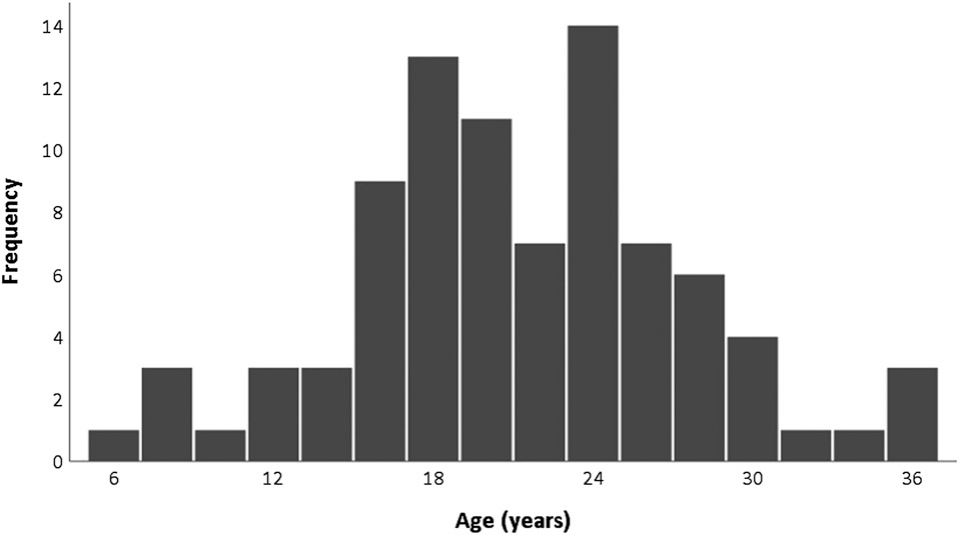
Age distribution of participants.

Figure 2 shows the age data, available for 87 of the 127 unique participants included in this review (Allen & Oyebode, 2009; Aslett et al., 2019; Gelman & Rhames, 2018; Hall & Sikes, 2017; Hutchinson, Roberts, Daly et al., 2016; Johannessen et al., 2015; Nichols et al., 2013). Ages ranged from 6 to 36 years (*M* = 20.86, *SD* = 6.35).

### Thematic synthesis

From the synthesis of included studies, 4 analytic themes and 11 subthemes were identified (Table 4).

**Table 4.** Summary of analytic themes and subthemes.

Analytic theme	Subtheme
Making sense of dementia	Change over time
	Comparisons to other illnesses
Impact of dementia	Emotional impact
	Caring responsibilities
	Roles and relationships
Coping	Difficulties coping
	Distancing
	Resilience
Support	Informal support
	Professional support
	Suggestions

#### Making sense of dementia

##### Change over time

Participants recalled noticing changes in their parents’ memory, mood, personality and behaviour. These were rarely attributed to the possibility of dementia but instead to stress, variations in mood, fatigue, menopause, distraction or different personality traits that the children were noticing as they matured (Millenaar et al., 2014; Sikes & Hall, 2018).

As changes became more apparent, medical attention was sought. Misdiagnosis and delays in accurate diagnosis lead to uncertainty and confusion. The diagnosis, often communicated via their other parent, was described as overwhelming, horrific, a shock and by some, a relief. It was important for children to know the diagnosis in order to understand their parents’ behaviour and attribute changes to illness (Nichols et al., 2013). One participant commented: ‘You have to be honest to kids, I think, they have a right to know, ‘cause if we do not…we will pick it up anyway’ (Svanberg et al., 2010, p. 742).

Participants discussed ongoing changes in their parent, including memory and communication difficulties and behavioural and personality changes, including withdrawal, disinhibition, aggression and changed interests and parenting practises. People found it particularly challenging if their parent was incontinent, aggressive or had forgotten who they were. They were constantly adapting to accommodate these changes, both practically and emotionally (Svanberg et al., 2010). ‘The need to keep “getting used to a new normal” did not get easier’ (Sikes & Hall, 2017, p. 332). However, despite a theme of disruption and distress, there was also a narrative of growth and coping (Gelman & Rhames, 2018).

The terminal nature of dementia was difficult to understand and accept. Eight articles mentioned the parent going into residential care or concern about this happening and the mixed emotions associated with this, including relief, sadness, worry and guilt. ‘The young people in these families seemed torn between relief at the easing of their care burden and sorrow that they had not been able to care for the fathers themselves’ (Allen & Oyebode, 2009, p. 471).

##### Comparisons to other illnesses

Dementia was often compared to other conditions, particularly cancer. There was a perception that it would be easier for their parent to have a condition that others understood, could empathise with, was curable and which did not affect cognition. One participant commented: ‘Whereas sometimes with other things, you’ve always got that little bit of hope but with Alzheimer’s that’s it’ (Hall & Sikes, 2017, p. 1208). People also spoke of the inequalities in research funding and support services for dementia compared to cancer (Hall & Sikes, 2017; Hutchinson, Roberts, Kurrle et al., 2016).

#### Impact of dementia

##### Emotional impact

Having a parent with dementia was experienced as incredibly sad, stressful and worrying. Participants described resentment (Allen & Oyebode, 2009), embarrassment of their parent (Hall & Sikes, 2017), envy of other children (Sikes & Hall, 2017) and anger and frustration regarding their situation (Johannessen et al., 2016), their parent’s behaviour (Millenaar et al., 2014) and the lack of acceptance by others (Hutchinson, Roberts, Kurrle et al., 2016). Feelings of shame, often resulting from discrimination, marginalisation and stigma, were common. Participants were distressed by rumours amongst peers (Nichols et al., 2013) and judgements of the public, which could lead to shame and secrecy (Hutchinson, Roberts, Daly et al., 2016).

Participants expressed guilt and self-blame, particularly when their patience wore thin (Nichols et al., 2013), and tried to avoid feeling guilty. One participant described their predicament as follows: ‘Sometimes I choose not to visit her, because then my whole day is spoiled. But then you have to go, or the feeling of guilt is even worse’ (Johannessen et al., 2016, p. 7).

They expressed sadness at their parent missing landmark events, such as winning awards, graduating, weddings and having children. People experienced loss and grief as their parent deteriorated, and confusion about losing their ‘real parent’: ‘It is almost like an in tandem place to be, you’re not bereaved, but you’re not *not* bereaved. You have a Dad but you have not got a Dad’ (Hall & Sikes, 2017, p. 1205). However, participants reported that their emotional well-being and life situations improved over time, since dementia onset (Johannessen et al., 2016). Five articles also discussed the positive impact, such as ‘pride in reciprocating care and supporting the family’ (Svanberg et al., 2010, p. 743) and feeling good about being able to help (Nichols et al., 2013).

##### Caring responsibilities

Children often prioritised their parents’ needs over their own. Responsibilities varied with age, but often included practical tasks such as cooking, supervising their parent, administering medication and communicating with professionals. Particularly challenging was supporting personal care. Many provided emotional support, supporting their parent’s self-esteem, cheering them up and maintaining their sense of being a valued member of society (Hutchinson, Roberts, Kurrle et al., 2016; Johannessen et al., 2016; Millenaar et al., 2014). One participant ‘felt anger towards everyone because of his or her lack of acceptance of her father with dementia and as a result was ready to fight for him to ensure he was not affected negatively by the discrimination she witnessed’ (Hutchinson, Roberts, Kurrle et al., 2016, p. 619). Participants also expressed concern for their healthy parent, noticing the increased responsibilities, stress and sadness and wanted to comfort and protect them, as well as their siblings.

The impact of caring responsibilities varied; some missed school or dropped out of college/university; however, others excelled and delved into academic and extracurricular activities (Gelman & Rhames, 2018). Some were less able to see friends, becoming socially isolated. Caring responsibilities impacted participants’ perceptions of the future, often changing their plans and decisions, including whether to go to university, career choices, relationships, starting a family and where to live. Uncertainty about the progression of dementia was difficult and some felt ‘a sense of “waiting” for their parent’s inevitable death, over an unknown period of time’ (Hall & Sikes, 2017, p. 1207) or feeling like life was on hold. Others avoided thinking about the future, instead taking each day as it comes (Allen & Oyebode, 2009). Despite these responsibilities, many did not view themselves as a young carer, minimising the significance of their caring role.

##### Roles and relationships

Dementia impacted the whole family. Many spoke of tension and conflict amongst family members and the importance of working together. Changes in family roles included the parent with dementia stopping work and the other parent working more for financial reasons or less to provide care (Allen & Oyebode, 2009). Some described the parent with dementia feeling more like a friend or developing a stronger relationship with them as a result of the shared caring experience (Nichols et al., 2013; Svanberg et al., 2010). Others emphasised the importance of them maintaining a parental role or criticised their other parent for ‘leaving the most responsible child to take on the caregiving work’ (Barca et al., 2014, p. 1939)*.* Extended family members were often perceived as unable to cope (Allen & Oyebode, 2009), not understanding (Gelman & Rhames, 2018) or neglecting (Hutchinson, Roberts, Daly et al., 2016).

Some felt that their parent with dementia had become disinterested and remote, leaving them feeling ignored and forgotten and losing their parent’s support in their own development (Barca et al., 2014; Johannessen et al., 2016; Svanberg et al., 2010). They often missed their old relationship (Hall & Sikes, 2017), feeling the need to ‘form a new relationship and accept the loss of the parent they knew before’ (Svanberg et al., 2010, p. 742). Participants often described a role reversal, whereby they were cast into a parental role. One participant, when talking about their parent, summed this up by saying: ‘she is my child, she really is’ (Johannessen et al., 2015, p. 250).

It often appeared helpful for participants to distinguish between dementia and their parent to cope with the changes. However, this was not always the case, and some used language that did not distinguish between the person and the illness. For example one participant commented: ‘It makes someone who was a lovely character really easy to dislike and you have to fight to not hate your own parent’ (Hall & Sikes, 2017, p. 1206).

#### Coping

##### Difficulties coping

Many spoke of how difficult it was to cope. Denial of reality was sometimes used as a way of coping (Allen & Oyebode, 2009). Some described struggles with depression, self-harm and thoughts of not wanting to be alive or of ending their life (Hall & Sikes, 2017). A combination of stressors, including bullying, moving to university, financial worries, their parent moving into residential care and lack of support from family and professionals contributed in making coping particularly difficult (Hall & Sikes, 2017; Hutchinson, Roberts, Daly et al., 2016). Concerns about burdening others led some to hide their difficulties, portraying that they were coping (Hutchinson, Roberts, Kurrle et al., 2016). Kevin commented: ‘There was lots of different things that I didn’t, I didn't really want to burden [Mum] with, that I’d bottle up’ (Svanberg et al., 2010, p. 744).

Other emotion-focused and avoidant coping strategies included using alcohol, drugs and smoking (Allen & Oyebode, 2009). Many found it difficult to speak about dementia (Hall & Sikes, 2017), sometimes due to believing that this would make them feel worse or be overwhelming (Johannessen et al., 2015; Millenaar et al., 2014). Others felt they had no one to speak to (Sikes & Hall, 2017).

##### Distancing

Participants often distanced themselves from their parent or the situation, needing to spend time away from the family home. This often led to improvement in the relationship with their parent and improvement in their own emotional well-being (Johannessen et al., 2016). For some, this physical escape could be extreme, as commented by 13-year-old Trudy: ‘I have memories of spending two nights in the elevator…because it was the warmest place in the winter’ (Hutchinson, Roberts, Kurrle et al., 2016, p. 617).

Coping sometimes required participants to detach emotionally or depersonalise their caregiving (Hutchinson, Roberts, Kurrle et al., 2016), which often had a negative impact on their emotional well-being.

Distancing by distraction or taking part in other activities (e.g. sport, choir and volunteering) was also common (Nichols et al., 2013). Many valued education and spending time with friends, enabling them to maintain a sense of normality. Some commented on how helpful it was that their friends were not going through the same thing (Hutchinson, Roberts, Daly et al., 2016).

##### Resilience

Over time, participants developed increasingly helpful coping strategies (Johannessen et al., 2016). Many reflected on the positive changes, such as becoming ‘more of a leader’, stronger, more mature or experiencing greater life satisfaction (Gelman & Rhames, 2018; Johannessen et al., 2016). One participant commented: ‘This happening to my father has inspired me in my academic life to excel…[and] to want to be a doctor…to help people like my Dad’ (Gelman & Rhames, 2018, p. 348).

Some people found it helpful to try to continue life as normal, watching TV, having family meals, going shopping or on holidays (Allen & Oyebode, 2009; Nichols et al., 2013). For younger participants, school could provide stability and a purpose, which was experienced as important and protective of their well-being (Hutchinson, Roberts, Daly et al., 2016). One 19-year-old participant commented: ‘You try to continue with your life as normal as possible without things influencing you’ (Millenaar et al., 2014, p. 2005). However, some found it unfair that others expected life to continue as normal, failing to appreciate the impact of dementia (Sikes & Hall, 2017).

Spending time with their parent and reminiscing about old memories could be helpful (Nichols et al., 2013); however, some found this upsetting (Johannessen et al., 2016). Many spoke of the importance of maintaining a positive but realistic attitude (Millenaar et al., 2014; Nichols et al., 2013), making the most of their situation, using humour and looking for positives (Svanberg et al., 2010). One participant gave the following advice: ‘My best advice to all those new to this situation is: use a lot of humour! You have much to gain!’ (Johannessen et al., 2016, p. 8). For others, turning to their faith was helpful.

#### Support

##### Informal support

Isolation and loneliness were common; participants reported that others either did not understand or had distanced themselves. Younger participants identified their parent without dementia as a main source of information and support (Nichols et al., 2013) and some were grateful to have a sibling to confide in (Allen & Oyebode, 2009; Hutchinson, Roberts, Daly et al., 2016). However, others felt neglected by family members, who failed to notice their distress (Hutchinson, Roberts, Kurrle et al., 2016) and experienced others as dismissive and invalidating.

Some participants sought support from friends, valuing having someone outside the family to talk to (Millenaar et al., 2014; Svanberg et al., 2010). However, others reported feeling that their peers were unsympathetic, ill-informed or did not want to deal with their difficulties, leading to reluctance in seeking their support (Gelman & Rhames, 2018; Hutchinson, Roberts, Daly et al., 2016). Older participants valued emotional and practical support from their partner (Barca et al., 2014).

##### Professional support

Some people received professional support through memory clinics (Millenaar et al., 2014), school, social services (Allen & Oyebode, 2009) or private arrangements. However, discussions often focussed on the lack of adequate and appropriate services. People felt unsure of where to get support (Hutchinson, Roberts, Daly et al., 2016), as dementia services were often aimed at older adults (Hutchinson, Roberts, Kurrle et al., 2016).

There was a dearth of resources and services designed for families of those living with young onset dementia, lack of information (Gelman & Rhames, 2018), absence of guidelines and recommendations (Barca et al., 2014; Nichols et al., 2013) and lack of understanding from professionals (Svanberg et al., 2010). As the child of the person living with dementia, they were often not consulted by professionals or invited to express their needs (Barca et al., 2014), resulting in them searching for information independently. Some had managed to find information online, which was helpful (Gelman & Rhames, 2018); however, others found the information overwhelming and not specific to their parents’ diagnosis (Millenaar et al., 2014).

Where services were available, families often had difficulties accessing this and had to actively seek it, describing this as a ‘battle’ and a ‘fight’ (Hall & Sikes, 2017; Johannessen et al., 2015), requiring them to ‘jump through hoops’ (Hutchinson, Roberts, Kurrle et al., 2016). Others did not feel able or know how to ask for support (Barca et al., 2014), sometimes due to stigma surrounding dementia and young carers (Hutchinson, Roberts, Kurrle et al., 2016). For those who were offered help, this either came too soon and was experienced as unnecessary, or was too late (Johannessen et al., 2015; Millenaar et al., 2014). One participant commented: ‘There is a need for it [support] but you should not have to ask for it yourself. It should be offered, because I would never have asked for it by myself’ (Barca et al., 2014, p. 1940).

Those who attended support groups generally reported finding this helpful (Barca et al., 2014; Gelman & Rhames, 2018; Hutchinson, Roberts, Daly et al., 2016), feeling less alone and more understood (Johannessen et al., 2016). However, many described difficulties finding support groups or found them too inclusive, for example they could be the only young person in the group (Barca et al., 2014). In one study, many commented that they did not feel in need of professional help, but some imagined needing this in future (Millenaar et al., 2014).

##### Suggestions

Participants made suggestions of what could be helpful for others caring for a parent living with young onset dementia. Many highlighted the need for education, including the importance of public knowledge and understanding. Some felt that information in school would be helpful, so teachers could facilitate children to access relevant support (Barca et al., 2014). People spoke of the importance of having someone to talk to who knew their parent and was familiar with their situation (Barca et al., 2014). They also had ideas to relieve burden, such as respite or befriending (Svanberg et al., 2010), and others suggested practical guidance on how to handle specific behaviours, such as stubbornness (Millenaar et al., 2014).

Support groups were often considered an important source of support (Gelman & Rhames, 2018; Svanberg et al., 2010); however, people highlighted the importance for these to be small and stratified by age (Nichols et al., 2013). Participants preferred face-to-face support but agreed that support via technology could be acceptable, and some (particularly teenagers) expressed interest in joining online forums (Barca et al., 2014; Nichols et al., 2013).

## Discussion

The aim of the current review was to systematically search, critically appraise and synthesise the qualitative literature regarding the lived experiences of those affected by parental young onset dementia. Data from 15 studies meeting criteria were appraised using the CASP qualitative checklist. Thomas and Harden’s (2008) three-stage approach for conducing thematic synthesis was followed, resulting in the organisation of data into four analytic themes and 11 subthemes.

All 15 studies meeting criteria were appraised and considered to be of adequate quality. However, overall quality varied. All studies used an appropriate research methodology, although detail regarding data analytic methods was mixed. Some provided a less detailed description of the ethical considerations (Millenaar et al., 2014; Nichols et al., 2013) and only two studies considered the relationship between researchers and participants (Sikes & Hall, 2017, 2018).

The themes captured the variety in people’s experiences of having a parent living with young onset dementia, with regards to ‘making sense of dementia’, the ‘impact of dementia’ on different aspects of their life, ‘coping’ and experiences of ‘support’.

Participants spoke about ‘change over time’ and the constant need to adapt to changing circumstances, a theme which has also been reported by adult children (aged 47–70 years) affected by parental late onset dementia (Hwang et al., 2017). Although children noticed change in their parent, dementia was generally not considered as a possible explanation, a finding possibly more specific to young onset dementia. Participants described the resulting long and confusing diagnostic process (Johannessen & Moller, 2013). Change over time continued as their parent’s health deteriorated, with many speaking about residential care and the difficult emotions associated with this. This desire to avoid institutional care has similarly been reported by those affected by parental late onset dementia (Hwang et al., 2017). Within the narratives, comparisons were drawn to other illnesses, especially those that are more common, less stigmatised or can be ‘cured’, such as cancer, with a sense that other illnesses would not have been as bad.

The impact of dementia varied, highlighting the need for a person-centred approach. Participants reported mixed emotions, although shock, sadness and grief were common. Many also mentioned positive emotions associated with caring, including pride. Caring responsibilities differed with age and circumstances and the impact of these responsibilities also varied. For example some became increasingly isolated or dropped out of education, whereas others excelled. The great sadness reported by participants, as a result of parents missing landmark events, may be particularly relevant for children and young adults, who are caring for their parent at a time when many of these events may be occurring.

Uncertainty for the future made it difficult for participants to plan ahead. Another subtheme, which has been previously reported by spouse caregivers, was changes in family roles and relationships (Sansoni et al., 2016). Many described the loss of their old relationship with the parent with dementia or role reversal, as well as changes in the relationship with their other parent.

The transactional model of stress and coping (Lazarus & Folkman, 1984) defines coping with stress as a process of ‘constantly changing cognitive and behavioural efforts to manage specific external and/or internal demands…appraised as taxing or exceeding [personal] resources’ (p.141). The participants in these studies often experienced stress and found coping difficult. Some unhelpful emotion-focused strategies were employed, such as denial and avoidance. However, these strategies could also be experienced as adaptive and protective. Those struggling to cope often experienced a combination of stressors. Other strategies, such as distancing and distraction, were mostly experienced as helpful. Using hobbies as a means to escape has similarly been reported by adult sons (aged 32–60 years) caring for a parent with dementia (McDonnell & Ryan, 2014). Some participants in the present review also reflected on the positive effects of caring and showed resilience, finding it helpful to attempt to continue life as normal or use humour to cope.

The main source of informal support was from immediate family and in particular the healthy parent and siblings. However, some felt isolated, dismissed or invalidated, particularly by extended family members. The conflict reported by adult children affected by parental late onset dementia is more often reported to be between siblings, for example over care matters (Hwang et al., 2017) or due to annoyance for siblings not being more involved (McDonnell & Ryan, 2014).

The amount of support received from friends also varied. Although some participants reported receiving professional support, this was often experienced as inadequate or inappropriate, and many found that as the child of the person living with dementia, support was not offered. Participants suggested that more information and support for children caring for a parent living with young onset dementia, such as small, age-specific support groups or access to online forums, would be helpful.

### Limitations

The process of quality appraisal is a subjective process, which is open to bias and interpretation. Although the CASP checklist is popular with qualitative researchers (Majid & Vanstone, 2018), it has been criticised for favouring articles that are sound with regards to compliance with expectations of research practice but make weaker contributions to the conceptual development of the field (Dixon-Woods et al., 2007).

The availability of qualitative studies looking specifically at the experiences of children under the age of 18 years was scarce. Many of the studies grouped children under the age of 18 years with adult children, and in most articles, the age range varied considerably, with some studies focussing exclusively on the experiences of adult children (e.g. Johannessen et al., 2015). Although it is likely that experiences vary by age, unfortunately it was not possible to report on these differences.

Eight of the 15 articles reported on the findings from the same three projects in the United Kingdom (Hall & Sikes, 2017, 2018; Sikes & Hall, 2017, 2018), Norway (Johannessen et al., 2015, 2016) and Australia (Hutchinson, Roberts, Daly et al., 2016; Hutchinson, Roberts, Kurrle et al., 2016). The decision was made to include all articles, as the focus of the articles differed, and an attempt was made to ensure that quotes were chosen from different sources.

Finally, although the included studies represented people from six different countries (USA, Canada, UK, Norway, Netherlands and Australia), these were all Western, high-income countries. It may be hypothesised that the experiences would vary depending on factors such as stigma, beliefs about dementia and cultural norms and expectations. Caution must, therefore, be applied when interpreting the findings.

### Clinical implications

The findings indicate a scarcity of appropriate support services to meet the needs of children of people living with young onset dementia and lack of information available for children regarding the diagnosis. In the first instance, it is crucial to raise awareness of young onset dementia amongst the public and professionals so that families feel more understood, more supported and less stigmatised. The findings also present the wide variations in individual experiences, highlighting the need for a person-centred approach. Changes are required in order to improve the diagnostic pathway and post-diagnostic support for people living with young onset dementia and their families, possibly through the introduction of more specialist services.

### Further research

This review highlights the paucity of public knowledge surrounding young onset dementia and appropriate interventions and support in place. Further research is required in order to broaden our understanding of young onset dementia and how best to support those affected.

## Conclusions

The current thematic synthesis presents the varied experiences of individuals affected by parental young onset dementia. There is evidently a lack of knowledge and understanding of young onset dementia by professionals and the public, and a scarcity of appropriate support. This, in combination with the stigma surrounding dementia and being a carer, can lead people to hide their difficulties.

These findings have important clinical implications for professionals working with families affected by young onset dementia and in particular those involved in service design and delivery. As the number of people being diagnosed with dementia is increasing and many of those living with young onset dementia are cared for by their children, it is important that further research is conducted to enable better understanding of and support for these families.
